# Supplementary material for: The effects of genital myiasis on the diversity of the vaginal microbiota in female Bactrian camels
Source: BMC Vet Res. 2022 Mar 5;18:87. doi: 10.1186/s12917-022-03189-5 (PMC8897907; doi:10.1186/s12917-022-03189-5)
Supplement: Supplementary file 5 — Additional file 5. [file 12917_2022_3189_MOESM5_ESM.zip › MPL201709200_16s_yy/Treat1/C01_diff/metastats/diff_group_genus.pdf]

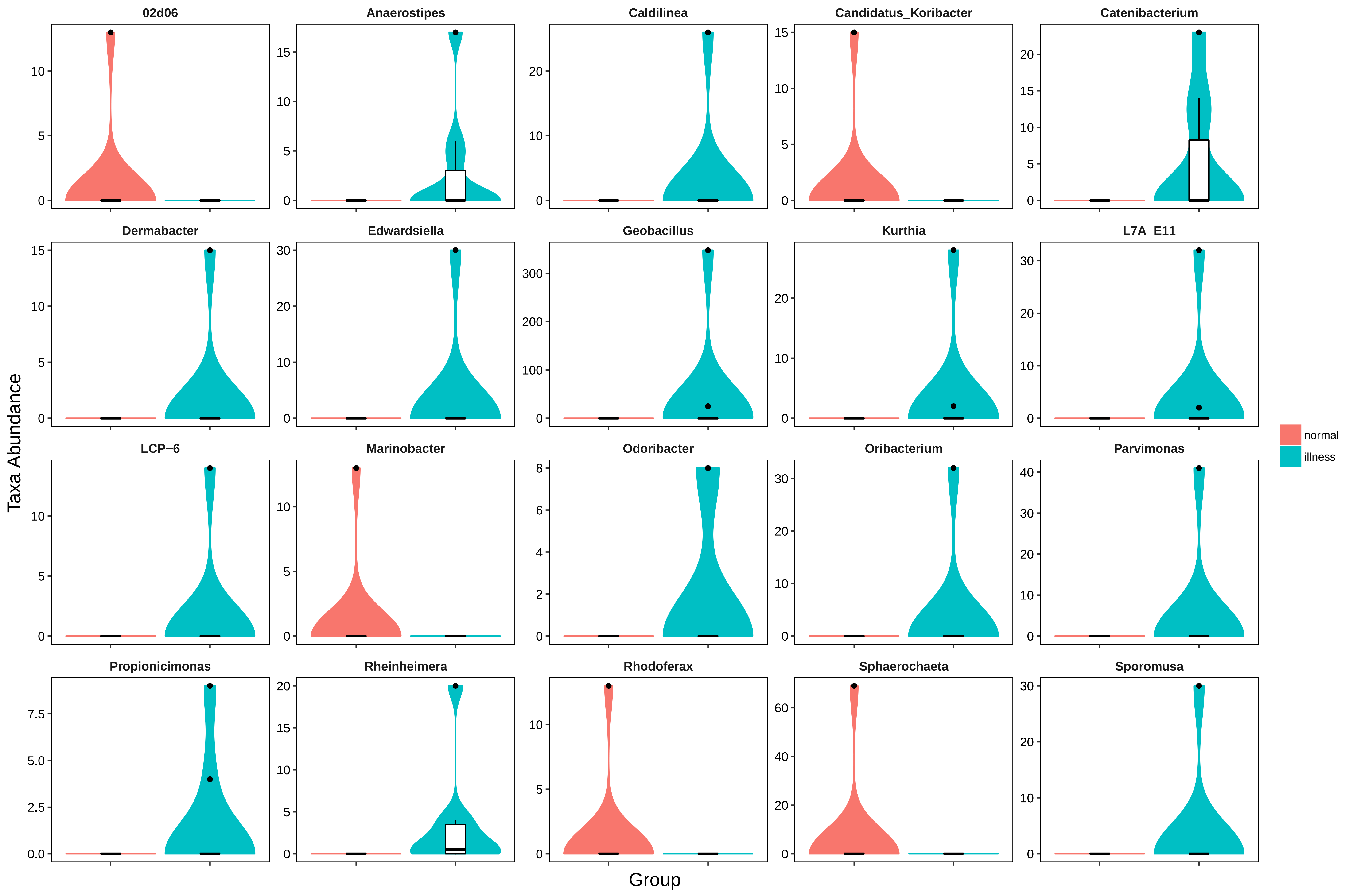

02d06
Anaerostipes
Caldilinea
Candidatus_Koribacter
Catenibacterium
Dermabacter
Edwardsiella
Geobacillus
Kurthia
L7A_E11
LCP−6
Marinobacter
Odoribacter
Oribacterium
Parvimonas
Propionicimonas
Rheinheimera
Rhodoferax
Sphaerochaeta
Sporomusa
Taxa Abundance
Group
normal
illness
